# Supplementary material for: Synthesis of Spherical Nanoparticle Hybrids via Aerosol Thiol-Ene Photopolymerization and Their Bioconjugation
Source: Nanomaterials (Basel). 2022 Feb 8;12(3):577. doi: 10.3390/nano12030577 (PMC8838805; doi:10.3390/nano12030577)
Supplement: Supplementary file 1 [file nanomaterials-12-00577-s001.zip › nanomaterials-1583846-SI for conversionupdate-done.pdf]

Supplementary Materials

Synthesis of Spherical Nanoparticle Hybrids Via Aerosol Thiol-Ene Photopolymerization and their Bioconjugation

Narmin Suvarli, Max Frentzel, Jürgen Hubbuch, Iris Perner-Nochta and Michael Wörner *

Department of Bio- and Chemical engineering, Institute of Process Engineering in Life Sciences, Section IV: Biomolecular Separation Engineering, Karlsruhe Institute of Technology, 76131 Karlsruhe, Germany; narmin.suvarli@kit.edu (N.S.); max.frentzel@gmx.net (M.F.); juergen.hubbuch@kit.edu (J.H.); iris.perner-nochta@kit.edu (I.P.-N.)

* Correspondence: michael.woerner@kit.edu

1. TEM Image of the Polymer Nanoparticle Composites

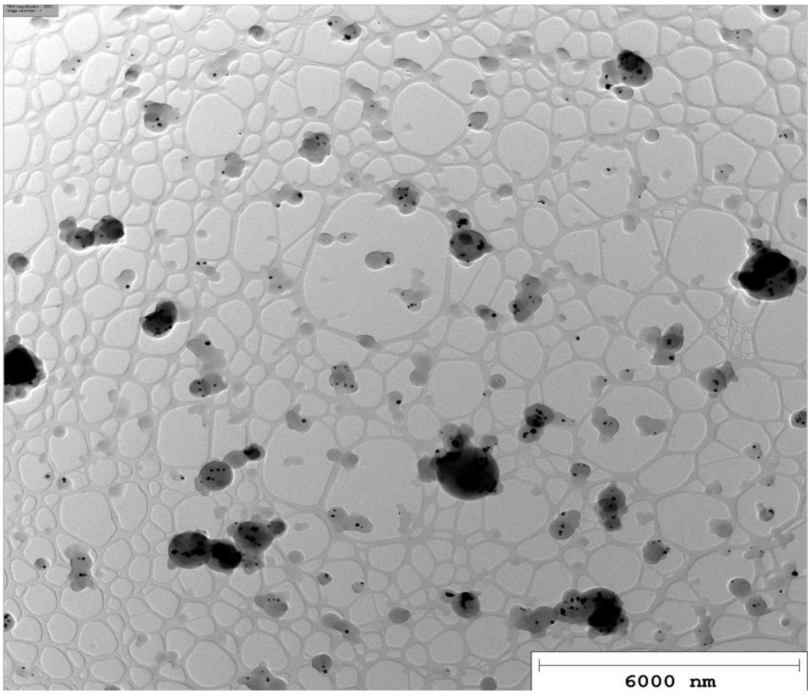

Figure S1. TEM image of NC-8 polymer nanoparticle hybrids on a lower magnification.

2. Size Distributions

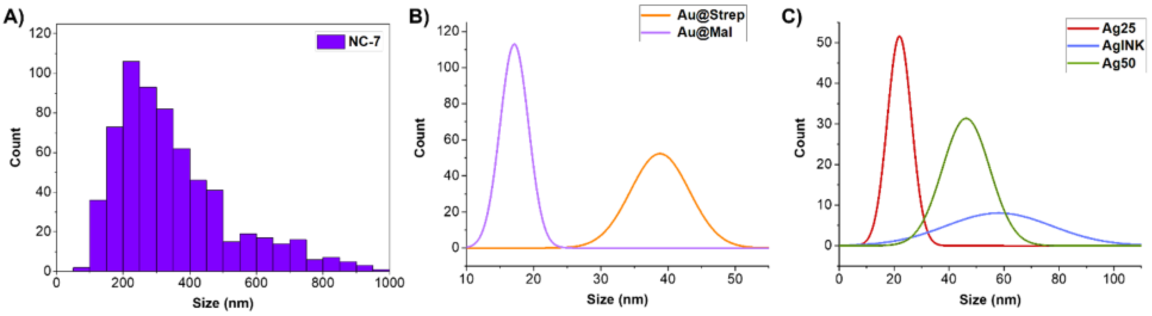

Figure S2. Size distributions of (**A**) polymer nanoparticle composites NC-7, (**B**) Au@Strep and Au@Mal nanoparticles and (**C**) Ag25, AgINK and Ag50 nanoparticles, as determined using image analysis of SEM and TEM micrographs.
